# Conjunctival Swabs Reveal Higher Detection Rate Compared to Schirmer Strips for SARS-CoV-2 RNA Detection in Tears of Hospitalized COVID-19 Patients

**DOI:** 10.3390/jcm11236929

**Published:** 2022-11-24

**Authors:** Luís Expedito Sabage, Young Joo Sun, Julian Wolf, Josmar Sabage, Alessandra Mazzo, Carlos Ferreira Santos, Vinit B. Mahajan, Luiz Fernando Manzoni Lourençone

**Affiliations:** 1 Bauru School of Dentistry, University of São Paulo, Bauru 17012-901, SP, Brazil; 2 Molecular Surgery Laboratory, Byers Eye Institute, Department of Ophthalmology, Stanford University, Palo Alto, CA 94304, USA; 3 Hospital for Rehabilitation of Craniofacial Anomalies, University of São Paulo, Bauru 17012-900, SP, Brazil; 4 Department of Ophthalmology, Lauro de Souza Lima Institute, Bauru 17034-971, SP, Brazil; 5 Veterans Affairs Palo Alto Health Care System, Palo Alto, CA 94304, USA

**Keywords:** SARS-CoV-2, COVID-19, tears, Brazil, dry eyes, comorbidity, prognosis

## Abstract

Purpose: To determine the prevalence of SARS-CoV-2 in tear samples and to investigate whether it correlates with ocular findings and patients’ prognosis in Brazil. Methods: Tears were collected using Schirmer strips (SS) and conjunctival swabs (CS) from patients hospitalized with laboratory-confirmed SARS-CoV-2 infection. Samples were analyzed using qRT-PCR. Demographic and clinical data, ocular symptoms, and Schirmer tests (ST) were collected from patients. Charlson Comorbidity Index (CCI) was used to rate comorbidities, and patients were monitored until hospital discharge or death. Results: There were 61 hospitalized patients, 33 of which were diagnosed with COVID-19. Within the confirmed COVID-19 patients, SARS-CoV-2 was detected in 18.2% (*n* = 6) of CS and 12.1% (*n* = 4) of SS samples. Subjective and objective parameters for dry eye syndrome (e.g., ST COVID-19: 8.3 ± 6.4mm, non-COVID-19: 8.9 ± 6.6mm, *p* > 0.05) were comparable between COVID-19 (*n* = 33) and non-COVID-19 patients (*n* = 28). Among the 16 COVID-19 patients exhibiting ocular symptoms, only tearing was reported significantly more frequently when tear samples were positive for SARS-CoV-2 (*p* < 0.05). Strikingly, patients whose tears tested positive for SARS-CoV-2 had significantly inferior CCI (pos.: 34.0 ± 31.8%, neg.: 67.6 ± 36.4%, *p* < 0.05) and higher mortality rates (pos.: 50.0%, neg.: 7.4%, *p* < 0.01). Conclusions: SARS-CoV-2 was detected with a prevalence of 18.2% on the ocular surface. Decreased CCI and increased mortality rate in the positive tear group suggests that viral detection may relate to prognosis and highlight the need of personal protective measures for healthcare professionals. Most of the patients, regardless of COVID-19 diagnosis, had low tear production and eye discomfort, possibly pointing to the need for artificial tear use during hospitalization.

## 1. Introduction

Since the onset of the pandemic in 2020, researchers have characterized COVID-19 symptoms as ranging from none to life threatening [1]. The most common symptoms are fever, cough, dyspnea, and sputum production [2,3]. Although eye symptoms are not often emphasized, light sensitivity, itchy eyes, tearing, eye redness, mucous discharge, foreign body sensation, and new onset floaters are symptoms that have been documented to occur in COVID-19 patients [4].

It is known that many respiratory viruses are present in the eye [5]. There are several factors that directly contribute to viral tropism, such as viral receptors in the host cell, the specific cell type, and physical barriers that enable and/or inhibit infections. Once inside a cell, the virus may damage or destroy it through direct cytopathic effects, host antiviral immune responses, and transformations of the infected cells that manifest as different ocular phenotypes [6]. Similar to other respiratory viruses, the main route for SARS-CoV-2 spread is human-to-human transmission from respiratory droplets that are expelled when coughing, sneezing, or even speaking [3]. Droplets typically travel 6 feet, however SARS-CoV-2 droplets can remain suspended in the air for as long as three hours [7]. The eye is in direct contact with the external environment, and it is connected to the respiratory system by the nasolacrimal duct, making it susceptible to pathogen contamination by droplets or even by direct contact with infected hands [8]. Angiotensin Converting Enzyme 2 (ACE2) and Transmembrane Serine Protease 2 (TMPRSS2), the key viral receptors for SARS-CoV-2 infection, are present on the ocular surface consisting of the cornea, conjunctiva, and limbus [9]. Although it is still unclear if these ocular surface receptors are sufficient to mediate clinical viral entry, there is evidence that the virus could be detected on the ocular surface, hence, tears may transmit the disease [10].

There are several methods to detect viruses on the ocular surface, with the most common being conjunctival swab (CS) sample collection followed by Polymerase Chain Reaction (PCR) or Reverse Transcription PCR (RT-PCR) analysis. Schirmer strips (SS) have shown different detection rates for different viruses because of how capillary flow dynamics, fluid viscosity, and gravity may affect the transfer of viral particles from tears to the filter paper [10,11]. Ocular surface samples collected by these two methods have different sample compositions: CS samples are composed of tears, cells, and fluids dispersed in the conjunctival sac, while SS samples consist of the substances and cells dissolved in tears [10].

SARS-CoV-2 detection in the eye has been investigated and a meta-analysis showed that ocular manifestations and positive SARS-CoV-2’s RNA were found, respectively, in 11.3% and 7.4% (0.0% to 57.0%) of patients with clinical- or laboratory-confirmed COVID-19 [12]. However, our understanding of SARS-CoV-2 and its ocular manifestation are limited. There are several regional variants of the virus with varying disease severities (each variant behaves uniquely, altering its pathogenic potential, severity, and symptoms), which might include differences in ocular manifestation [13]. Of these clinical COVID-19 studies, ocular symptoms were subjectively evaluated, hence the results showed a heterogeneity of 80.3% due to the lack of protocols for data collection [12]. In addition, the impact of comorbidities and patient’s prognosis in association with ocular symptoms has not been studied. It is known that older patients with more comorbidities tend to have more severe forms of the disease, and thus a poor prognosis and higher mortality rate. Charlson Comorbidity Index (CCI) is a validated questionnaire that predicts patients’ prognosis (10-year mortality rate), and it is currently the best predictor for COVID-19-hospitalized patients [14]. The objective of this study is to determine the prevalence of SARS-CoV-2 in tear samples and to assess whether the viral detection in ocular surface samples correlates with ocular findings and patient’s prognosis.

## 2. Materials and Methods

### 2.1. Study Design

A cross-sectional study was conducted at The Clinics Hospital of Bauru, University of São Paulo, the reference center for COVID-19 treatment in Bauru’s health division, São Paulo, Brazil, from July 2021 to November 2021. Although SARS-CoV-2 strains were not directly identified in this study, it should be noted that circulating variants in the state of São Paulo, Brazil, were mainly Gamma (P.1 and P.1.*) and Delta (AY.99.2, AY.101, and AY.*+B.1.617.2) during this study period [15].

The study was designed to analyze COVID-19 prevalence in the tears of the hospitalized patients, where study data can be collected under a more controlled environment. Tear samples were collected from adult patients at least 18 years of age, hospitalized with laboratory-confirmed COVID-19, and displayed moderate flu-like syndrome and/or mild-to-moderate acute respiratory distress syndrome (ARDS). Patients who received a negative COVID-19 testing result from a real time quantitative PCR (qRT-PCR) nasopharyngeal swab test were included as a control cohort for comparison of Schirmer Test values to address the prevalence of low tear production in the COVID-19 and non-COVID-19 patients. Before ocular surface sample collection, a patient questionnaire on eye symptoms was conducted to inquire about their ocular surgery history, use of contact lenses, and use of eye drops. Patients were asked if they had one of the following symptoms in the past seven days: tearing, foreign body sensation, pain, and red eye, which was confirmed through ectoscopy. This questionnaire was built to be a short, fast anamnesis in ocular symptoms that could summarize ocular discomfort and factors related to ocular surface changes. We acknowledge that no validated questionnaires to address dry eye syndrome were used in this study. Demographic data (e.g., age and sex), vaccine history, and the CCI were collected from medical records. CCI was calculated using an online tool (“https://www.mdcalc.com/charlson-comorbidity-index-cci” accessed on 5 March 2022). The score includes the following parameters: age, myocardial infarction, congestive heart failure, peripheral vascular disease, cerebrovascular accident, dementia, chronic obstructive pulmonary disease, connective tissue disease, peptic ulcer disease, liver disease, diabetes mellitus, hemiplegia, chronic kidney disease, solid tumor, leukemia, lymphoma, and AIDS. After two months of the sample collection, all medical records were revised for hospital discharge date or death.

### 2.2. Tear Sample Collection

**Figure 1:**
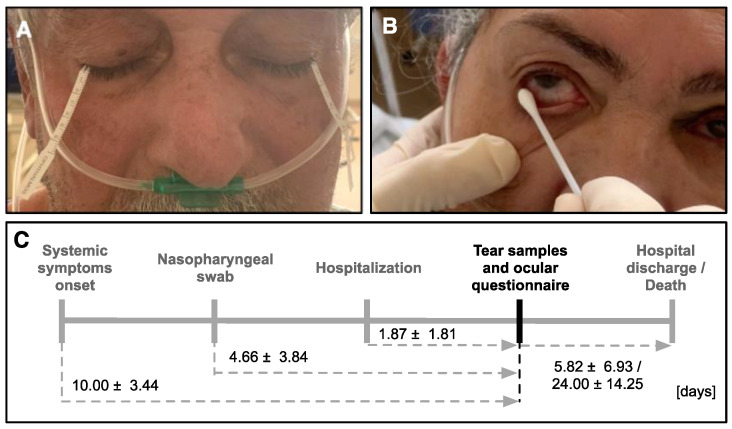
Samples were collected using Schirmer strips and conjunctival swab after hospitalization, and patients were followed until discharge or death. (**A**) Collection of Schirmer strips. (**B**) Collection of conjunctival swab. (**C**) Overview of timepoints of sample collection. Numbers indicate mean days and standard deviation between the indicated event and tear sample collection.

All patients were placed in a sitting position without ocular anesthesia and samples were collected by a single researcher wearing full personal protective equipment as recommended by the Centers for Disease Control and Prevention (CDC). With a sterile surgical glove, SS (Whatman #41 0.2 × 5.0 × 60.0 mm filter paper; Ophthalmos S.A.^®^, São Paulo, SP, Brazil) were folded 5 mm from one end and placed in the inferior temporal canthus of each eye for 5 min (Figure 1A); patients were asked to keep their eyes closed during the test. Both strips were removed with a sterile clamp, the value of the wet part was written down in millimeters (Schirmer test), and the SS were inserted in one 15 mL centrifuge tube (Falcon^®^, Corning, NY, USA) with a 600 μL of 0.9% saline solution. From 1 to 2 min after SS, CS were then collected in the inferior palpebral conjunctiva in two rolling movements from temporal canthus to nasal canthus, and a different swab was used for each eye (Figure 1B). Both CS were inserted in one 50 mL centrifuge tube (Falcon^®^, Corning, NY, USA) containing a 600 μL of 0.9% saline solution. Samples were immediately sent to the laboratory for qRT-PCR and analyzed immediately for SARS-CoV-2 viral gene detection for diagnostic purposes. Samples were collected in both eyes and pooled to increase the total volume of sample. The literature shows one case report where SARS-CoV-2 was detected unilaterally, but this patient had an obstruction of the common lacrimal duct, contributing to a lower rate of tear washing and, thus, a higher chance of viral detection in that eye [16].

### 2.3. SARS-CoV-2 Viral Genome Detection by Quantitative Real-Time Polymerase Chain Reaction (qRT-PCR)

Viral RNA isolation and purification for diagnostic criteria was performed using Extracta Kit Fast-DNA and RNA (MVXA-P096 FAST) (Loccus®, Cotia, Brasil) according to manufacturer’s protocol in an automatized extractor Extracta 96 (Loccus^®^, Brasil). For mRNA fragments amplification of Nucleocapside (*N*), Envelope (*E*), and RNA-dependent RNA polymerase (*RdRp*) genes, GeneFinder COVID-19 Plus RealAmp Kit (Osang Healthcare^®^, Anyang, Republic of Korea) was used. Human RNAseP was used as a positive control for RNA extraction. Reverse Transcription was performed simultaneously in a single step. For PCR composition, in a 384 well plate, 5 μL of extracted RNA, 10 μL of COVID-19 Plus Reaction Mixture, and 5 μL of COVID-19 Plus Probe Mixture (COVID-19 Plus RealAmp Kit, Osang Healthcare^®^, Republic of Korea) were mixed. For each test, a negative control was performed with distilled water. The plate was inserted in the real time PCR equipment Viia 7 (Applied Biosystems) with the following thermal cycling configuration: (1) 20 min at 50 °C (for reverse transcription); (2) 5 min at 95 °C (for inactivation of reverse transcriptase and activation of Taq polymerase); and (3) 45 cycles of 15 s at 95 °C and 1 min at 58 °C (for amplification). Results were analyzed based on cycle threshold (Ct) values to determine SARS-CoV-2 positivity following manufacturer’s instructions (GeneFinder™ COVID-19 Plus RealAmp Kit (Osang Healthcare^®^, Republic of Korea)), and were determined to be negative when Ct values were superior to 41.

### 2.4. Statistical Analysis

Statistical analyses were performed using IBM SPSS^®^ statistical software version 28.0.1 (IBM, Inc., Chicago, IL, USA). Continuous variables were compared between groups with Mann–Whitney U and Kruskal–Wallis test for non-parametric independent samples. Two continuous variables were correlated using Kendall’s tau b test and nominal variables were compared with Chi-squared test.

### 2.5. Ethical Aspects

The study was performed in accordance with the ethical standards of The Declaration of Helsinki and approved in the Local Research Ethics Committee (Hospital of Rehabilitation in Craniofacial Anomalies-University of São Paulo, Bauru, SP, Brazil) under number 4.052.064. All patients signed an informed consent agreement before sample collection.

## 3. Results

### 3.1. Patients’ Demographics

Tear samples were collected from 61 hospitalized patients in a Brazil hospital. Based on the qRT-PCR nasopharyngeal test, 28 patients tested COVID-19 negative, and 33 patients were positive. Age (58.6 ± 18.4 years (range 23 to 87) vs. 59.4 ± 16.5 (range 24 to 86), *p* = 0.994) and sex (male: 57.6% vs. 42.9%, *p* = 0.252) were comparable between COVID-19 positive and negative patients, which was also generally representative of the demographics for hospitalized patients in Brazil [17]. These COVID-19 patients were hospitalized with a broad spectrum of systemic symptoms (Supplemental Table S1), which were already reported as possible COVID-19 symptoms [18]. Before sample collection, most patients required oxygen support (72.7%), but it was provided only by nasal cannula, and no other mechanisms were used.

The most prevalent comorbidities were systemic arterial hypertension (48.5%), diabetes mellitus type 2 (24.2%), obesity (24.2%), and dyslipidemia (12.1%). Other comorbidities were also found in lower prevalence (Supplemental Table S2). Charlson Comorbidity Index (CCI) of COVID-19 positive patient cohort was 62.6% ± 37.8 (range 0% to 98%) while the COVID-19 negative patient cohort was 55.0% ± 40.7% (range 0% to 98%, *p* = 0.319; Table 1). Notably, each group had a similar ratio of unvaccinated patients—38.8% and 35.7% for positive and negative cohorts (vaccine status was only available for 14/28 non-COVID-19 patients), respectively. Five COVID-19 positive patients (15.2%) expired 24.0 ± 14.2 days (ranging 7 to 41 days) after sample collection, while none died in the negative cohort. The collection timeline is shown in Figure 1C. Notably, the expired patients had a longer hospitalization period (24.5 ± 13.9 days) compared to discharged patients (7.9 ± 6.6 days).

### 3.2. SARS-CoV-2 Was Better Detected in Tear Samples Collected by Conjunctival Swab Compared to These by Schirmer Strips

**Figure 2:**
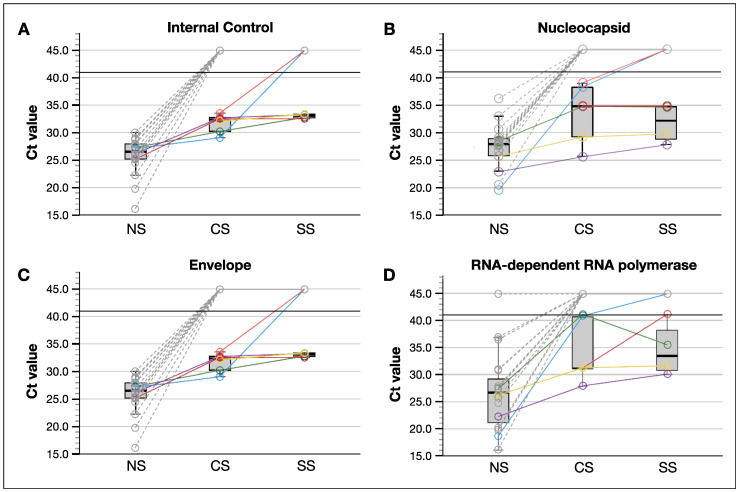
Nasopharyngeal swabs had lower cycle threshold values than tear samples. (**A**) Internal control. (**B**) Nucleocapsid. (**C**) Envelope. (**D**) RNA-Dependent RNA Polymerase. Colored and grey lines represent, respectively, patients with a positive and negative detection of SARS-CoV-2 in tear samples, each different colored line represent the same patient. Black line shows the cut off value for test positivity. Each dot corresponds to one patient. CS: conjunctival swab; E: envelope; IC: internal control; N: nucleocapsid; Na: nasopharynges; RdRp: RNA-dependent RNA polymerase; SS: Schirmer strip.

SARS-CoV-2 viral RNA positivity was determined by cycle threshold (Ct) values for nucleocapsid (*N*), envelope (*E*), and RNA-dependent RNA polymerase (*RdRp*) (Figure 2). Tears were analyzed in a total of 47 patients: 33 COVID-19 positive and 14 patients from the 28 COVID-19 negative patients. As expected, none of the COVID-19 negative patients (*n* = 14) determined by nasopharyngeal swab (NS) had a positive tear sample. For the tear samples of COVID-19 positive patients (*n* = 33), SARS-CoV-2 RNA was detected in 6 (18.2%) and 4 (12.1%) tear samples collected by CS and SS, respectively. Every patient who had a COVID-19 positive SS sample also had a positive CS sample. Interestingly, there was a trend towards lower Ct values in CS samples compared to SS samples, however the difference was not significant. These results indicate that CS may represent a better sample collection method to detect viral genes in tears.

We compared the Ct values of tear samples to these of matching nasopharyngeal swab samples from 19 COVID-19 positive patients: 14 patients in our COVID-19 positive cohort were excluded, because we did not have access to the Ct values from nasopharyngeal test taken before their hospitalization. We found that tear samples had significantly higher Ct values compared to the nasopharyngeal swabs (31.7 ± 1.7 vs. 25.9 ± 3.4, *p* < 0.001; Figure 2), indicating that the tear samples contained significantly less viral genes compared to the nasopharyngeal samples.

Notably, only the tear samples collected by CS within four days after nasopharyngeal sample collection contained a significant load of viral genes to be tested positive. Every patient with positive CS tear sample had a Ct value below 30 from nasopharyngeal swab testing, suggesting that tear sample collection timing and relatively higher viral load in patient (nasopharyngeal Ct value < 30) may affect SARS-CoV-2 detection in tears.

### 3.3. Patients Who Had SARS-CoV-2 Positive Tear Samples Reported Significantly More Subjective Ocular Symptoms, However Objective Measurements Did Not Differ

There were 16 (48.5%) of the COVID-19 positive patients and 12 (42.9%) of the negative patients who reported ocular symptoms, including foreign body discomfort (10 and 4 patients in each group), pain (4 and 2), red eye (4 and 5), and tearing (3 and 2) (*p* > 0.05 for all symptoms, chi-squared test). Within the 33 COVID-19 positive patient cohort, patients with SARS-CoV-2 positive tears reported significantly more tearing symptoms compared to the patients with SARS-CoV-2 negative tears (*p* = 0.022; Table 2). Of the COVID-19 positive patients, 24.2% already had had previous eye surgery—6 had cataract surgery in both eyes, 1 had cataract surgery and vitrectomy in both eyes, and 1 had pterygium surgery in both eyes. Reported symptoms did not increase significantly in patients with previous eye surgery when compared to patients without (*p* = 0.632). None of the patients reported the use of contact lenses or eye drops.

Schirmer tests (ST) were used to objectively analyze tear production and dry eye. The prevalence of dry eye syndrome—as defined by a ST value below 10 mm after 5 min, [19]—was comparable between COVID-19 positive (57.7%) and negative patients (64.2%, *p* = 0.593), as well as between COVID-19 patients with positive (66.7%) or negative (59.3%, *p* = 0.874) SARS-CoV-2 tear detection. Accordingly, there was no statistical difference (*p* = 0.706) in ST results between COVID-19 patients and non-COVID-19 patients (8.3 ± 6.4 mm (range 1 to 25) and 8.9 ± 6.6 mm (range 1 to 25)), respectively. Similarly, there was also no statistical difference in ST results between patients with SARS-CoV-2 negative tears and positive tears (8.3 ± 6.3 mm (range 1 to 25) and 8.3 ± 7.5 mm (range 1 to 21), respectively; Supplemental Figure S1). These findings suggest that SARS-CoV-2 may not be a significant factor that could cause dry eye syndrome in hospitalized patients. Instead, disregarding a patient’s SARS-CoV-2 positivity, ST was negatively correlated to age (*p* = 0.021, R = –0.209) and positively correlated with CCI (*p* = 0.014, R = 0.230), showing that older patients with more comorbidities had drier eyes.

### 3.4. Patients with SARS-CoV-2 Positive Tears Had Worse Prognosis

Out of our 33 COVID-19 positive patient cohort, 5 expired. As expected, most of the expired patients had a low estimated 10-year survival rate based on their Charlson Comorbidity Index (CCI). Notably, each patient who expired was fully vaccinated with CoronaVac (at least two doses), and they all had three or more comorbidities (Supplemental Table S3).

**Figure 3:**
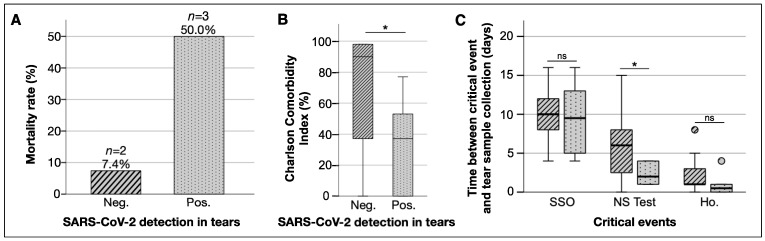
SARS-CoV-2 detection in tear samples is associated with mortality rate, Charlson Comorbidity Index, and sample collection timing. (**A**) Patients who had viral particles detected in tear samples had a higher mortality rate. (**B**) Boxplot comparing CCI and SARS-CoV-2 detection in tear samples. (**C**) Boxplot comparing critical periods of sample collection. Timing represents the number of days between tear collection and the events on the *X*-axis. *: *p* < 0.05. Ho.: hospitalization; Neg.: negative SARS-CoV-2 in tear samples; NS: nasopharyngeal swab; Pos.: positive SARS-CoV-2 in tear samples; SSO: systemic symptoms onset.

From the 5 expired patients, 3 of them had a SARS-CoV-2 positive tear sample, while the tear samples from the other 2 patients were negative. These results indicate that patients with a positive SARS-CoV-2 tear sample had a mortality rate of 50%, whereas the mortality rate was only 7.4% for those with a negative tear sample (*p* = 0.008; Figure 3A). In addition, the CCI was significantly lower in expired patients with a positive tear test compared to the ones with a negative tear test (*p* = 0.027; Figure 3B). However, positive tear testing was not significantly associated with lower CCI scores when also considering the non-fatal COVID-19 patients (*p* = 0.058). Additionally, the number of days since systemic symptom onset and hospitalization had no influence on tear detection (Figure 3C). Taken together, these findings suggest that patient comorbidity may be a dominant factor over SARS-CoV-2 positivity in tear samples for COVID-19 patient prognosis, yet SARS-CoV-2 positivity in tear samples can be an indirect indicator for poorer COVID-19 patient prognosis.

## 4. Discussion

Our study shows that it is possible to detect SARS-CoV-2 RNA in tear samples collected either by conjunctival swab (CS) or Schirmer strips (SS) using qRT-PCR. However, the prevalence of SARS-CoV-2 detection in tear samples was low (18.2% in CS and 12.1% in SS samples from laboratory-confirmed COVID-19 patients). Viral detection in tear samples has been explored in several other systemic viruses, including SARS-CoV [20,21], Zika [22], Adenovirus [23,24,25], and Hepatitis B [26]. In these previous studies, the prevalence of a virus in tears varied based on the genetic material of the virus—a DNA virus had a higher prevalence compared to RNA virus (Supplemental Figure S2). SARS-CoV-2 prevalence in tears has already been explored in other studies, ranging from 0% to 57% [27,28]. Most of these studies used only CS for tear collection [27,29,30,31,32,33,34,35,36,37,38,39,40,41,42], one study used only SS [43], and four other studies used both tear collecting methods [44,45,46,47]. Our study confirms previous findings that CS samples had a higher SARS-CoV-2 detection rate compared to SS samples, and the viral loads in tear samples were lower compared to nasopharyngeal samples [44,45,46,47]. This is possibly because conjunctival scratching permits the collection of conjunctival cells, resulting in a larger volume of samples and, consequently, higher positivity rates. Therefore, tear sample collection using conjunctival swab might be a more preferred method to detect the presence of SARS-CoV-2 on the ocular surface rather than Schirmer strips.

In contrast to previous studies in which *N* and *RdRp* viral genes were not considered in the qRT-PCR analyses, our study enabled the detection of different parts of the virus, which yielded an improved detection rate since more samples showed positive results for *N* (Figure 2). In addition, most previous SARS-CoV-2 in tear studies were conducted before any SARS-CoV-2 variants emerged, and only two studies were conducted during the time and location where the Alpha variant circulated. Importantly, our study was conducted in the state of São Paulo, Brazil, during the period when circulating variants were mainly Gamma (P.1 and P.1.*) and Delta (AY.99.2, AY.101, and AY.*+B.1.617.2) [48].

The time between tear sample collection and systemic symptom onset was on average, 10 days regardless of COVID-19 positive or negative status in the tear samples. Patients with positive tear samples had a significantly shorter window between the nasopharyngeal swab and tear collection, indicating that viral RNA in tear samples might be more prevalent in the earlier stages of infection. However, nine other patients also had the tear collection before 4 days and tested negative, and the only difference between them was CCI. In Brazil, before entering a tertiary hospital, patients with clinical symptoms of COVID-19 were evaluated in less complex assistance units where they received initial assistance, orientation, and were—if necessary—referred to a tertiary hospital. This process influenced the results timeline. Since the number of days from hospitalization was a controllable factor, the study was designed to analyze the prevalence of COVID-19 in the tears of hospitalized patients. Severe patients were not included because they tended to present more confounders (e.g., high dose medications, invasive oxygen support, and longer periods in the hospital). However, a limitation of this and other COVID-19 studies is the time of exposure to SARS-CoV-2 before disease onset, a variable which cannot be definitively confirmed. The date of symptom onset is subjective because it depends on a patient’s ability to recognize symptoms and report them accurately. Reporting is also arbitrary because symptom thresholds are different between individuals, and disease phenotypes gradually increase in the first days of the infection [49].

Interestingly, in this study, patients who had SARS-CoV-2 particles detected in tear samples had an almost sevenfold increased chance of dying compared to patients with a negative result. Those patients also had other factors that could contribute to death, such as a 10-year poor survival rate calculated by CCI. Viral detection in tear samples was not correlated with longer hospitalizations. This suggests that patients with higher viral loads and disseminated viremia might have poorer prognosis, [50,51] and, thus, a higher chance of viral detection in different fluids. This point is meaningful because healthcare professionals should be aware while taking care of COVID-19 patients, especially those with more comorbidities, of higher viral loads in nasopharyngeal swabs and a worse prognosis, since tears might have a potential to be a fluidic transmitter of virus via ocular or non-ocular routes. Additionally, viral detection in tears may potentially serve as an indicator for a moderate disease becoming severe, since most patients died even with a stable and non-severe clinical condition at the time of collection. These points are still gaps of knowledge, and more research on this is needed to clarify prognosis and which patients to be more aware of while in a normal hospital infirmary or intensive care unit.

Almost half of the patients in the study reported at least one ocular symptom, but COVID-19 does not have pathognomonic features and ocular findings that could relate to a broad variety of diseases and conditions, including dry eye syndrome, corneal abrasions, and trauma. None of the patients reported trauma or showed signs of ocular infections, even though hospitalization is known to cause ocular discomfort because of medications, oxygen devices, and stress [52]. The only statistically significant symptom between the patients whose tears contained SARS-CoV-2 and those that did not was tearing. Although patients reported tearing, when the volume of tears was objectively analyzed through ST, there was no difference between groups. However, subjective tearing may be a sign of tear film instability, which is not tested by ST, but could be evaluated in further studies using the tear breakup time test [53]. It could be possible that the patients who reported tearing were having eye discomfort because of SARS-CoV-2 infection, and thus they reported this discomfort as tearing. However, the symptoms were nonspecific and did not relate to any other findings. There was also no statistical difference between ST in COVID-19 and non-COVID-19 patients in the tertiary care hospital. However, both groups presented a low average test, suggesting compromised tear film quality and pointing to the potential need to use artificial tears to prevent and/or treat dry eye syndrome and ocular surface damage in hospitalized patients. Multicentric studies with larger cohort size are needed to better address how to prevent degradation of tear film quality, how to restore tear film quality during hospitalization, and how to maintain tear quality health after hospital discharge in COVID-19 patients considering comorbidities, time of hospitalization, and oxygen use.

We acknowledge that this study is limited by a relatively small sample size (33 confirmed COVID-19 and 28 control patients). In addition, no complete ophthalmological examination was performed (only direct visualization of the external parts of the eye). It was not possible to take infected COVID-19 patients to the ophthalmology exam room, and no portable devices were available.

Even though the virus could be detected in tears, it remains unclear if the ocular surface could be an infection site. Previous postmortem studies showed that SARS-CoV-2 can be detected in ocular surface tissues [54] and even continue replicating on the ocular surface even after death (cadaver and eye organoid cultures) [55]. These findings could be a concern for cornea transplantation, postmortem studies, and ophthalmology practice, however this question has yet to be addressed [56].

## 5. Conclusions

This study showed that SARS-CoV-2 is detectable in tears, especially in patients with more comorbidities. Since a potential transmission of SARS-CoV-2 from tears through ocular and non-ocular routes cannot be excluded, healthcare professionals should be aware while taking care of COVID-19 patients, especially those with more comorbidities, higher viral loads in nasopharyngeal swab, and a worse prognosis.

## Figures and Tables

**Table 1:** Patients’ demographic.

	COVID-19			Non-COVID-19
	Total	SARS-CoV-2 Detection in Tears	No SARS-CoV-2 Detection in Tears	Total
Number of Patients	33	6	27	28
Sex				
Male	57.6% (19)	33.3% (2)	62.9% (17)	42.9% (12)
Female	42.4% (14)	66.7% (4)	37.1% (10)	57.1% (16)
Mean age (range)	58.6 (23–87)	68.7 (56–82)	56.3 (23–87)	59.4 (24–86)
Mean CCI (range)	62.6% (0.0–98.0%)	34.0% (0.0–77.0%)	67.6% (0.0–98.0%)	55.0% (0.0–98.0%)
SAH	48.5% (16)	83.3% (5)	40.7% (11)	39.3% (11)
DM2	24.2% (8)	50.0% (3)	18.5% (5)	17.8% (5)
Obesity	24.2% (8)	33.3% (2)	22.2% (6)	25.0% (7)
Dyslipidemia	12.1% (4)	16.7% (1)	11.1% (3)	3.6% (1)
Vaccine				
CoronaVac 3rd dose	6.6% (2)	33.3% (2)	0.0% (0)	0.0% * (0)
CoronaVac 2nd dose	36.4% (12)	50.0% (3)	33.3% (9)	42.9% * (6)
CoronaVac 1st dose	9.1% (3)	0.0% (0)	11.1% (3)	0.0% * (0)
Oxford’s AZD1222 2nd dose	0.0% (0)	0.0% (0)	0.0% (0)	7.1% * (1)
Oxford’s AZD1222 1st dose	9.1% (3)	0.0% (0)	11.1% (3)	14.3% * (2)
No vaccine	38.8% (13)	16.7% (1)	44.5% (12)	35.7% * (5)
Unknown	0	0	0	14
SARS-CoV-2 testing in tears (conjunctival swab and Schirmer strips)	100.0% (33)	100.0% (6)	100.0% (27)	50.0% (14)

CCI: Charlson Comorbidity Index; DM2: Diabetes Mellitus type 2; SAH: systemic arterial hypertension. *: the percentage refers to the group of patients with known vaccination status.

**Table 2:** Ocular symptoms were reported on high frequency.

	Total (33)	SARS-CoV-2 Tear Samples		χ^2^
		Positive (6)	Negative (27)	
Reported ocular symptoms	48.5% (16)	50.0% (3)	48.1% (13)	ns
Foreign body sensation	30.3% (10)	16.6% (1)	33.3% (9)	ns
Tearing	9.1% (3)	33.3% (2)	6.9% (1)	0.022
Red eye	12.1% (4)	16.6% (1)	11.1% (3)	ns
Pain	12.1% (4)	0.0% (0)	14.8% (4)	ns
Previous eye surgery	21.2% (7)	16.6% (1)	22.2% (6)	ns
Use of eye drops	0.0% (0)	0.0% (0)	0.0% (0)	ns
Use of O_2_ (nasal cannula)	72.7% (24)	100.0% (6)	66.6% (18)	ns

Ocular questionnaire results. All questions were asked before sample collection, and reported symptoms were only considered if they started within the last 7 days. ns: not significant.

## Data Availability

Direct information and requests for resources or data to Luiz F. M. Lourençone (luiz.fernando@usp.br).

## Supplementary Materials

The following supporting information can be downloaded at: https://www.mdpi.com/article/10.3390/jcm11236929/s1, Figure S1: Schirmer test was not significantly different between COVID-19 and non-COVID-19 patients; Figure S2: Systemic viruses show different detection prevalence in tear samples; Table S1: Systemic symptoms in the confirmed COVID-19 group; Table S2: Older patients also showed different comorbidities; Table S3: Patients who died were fully vaccinated, however they had three or more comorbidities.
