# Supplementary material for: Conjunctival Swabs Reveal Higher Detection Rate Compared to Schirmer Strips for SARS-CoV-2 RNA Detection in Tears of Hospitalized COVID-19 Patients
Source: J Clin Med. 2022 Nov 24;11(23):6929. doi: 10.3390/jcm11236929 (PMC9737489; doi:10.3390/jcm11236929)
Supplement: Supplementary file 1 [file jcm-11-06929-s001.zip › jcm-2031311-supplementary.pdf]

Journal of
Clinical Medicine

MDPI

Supplementary Materials

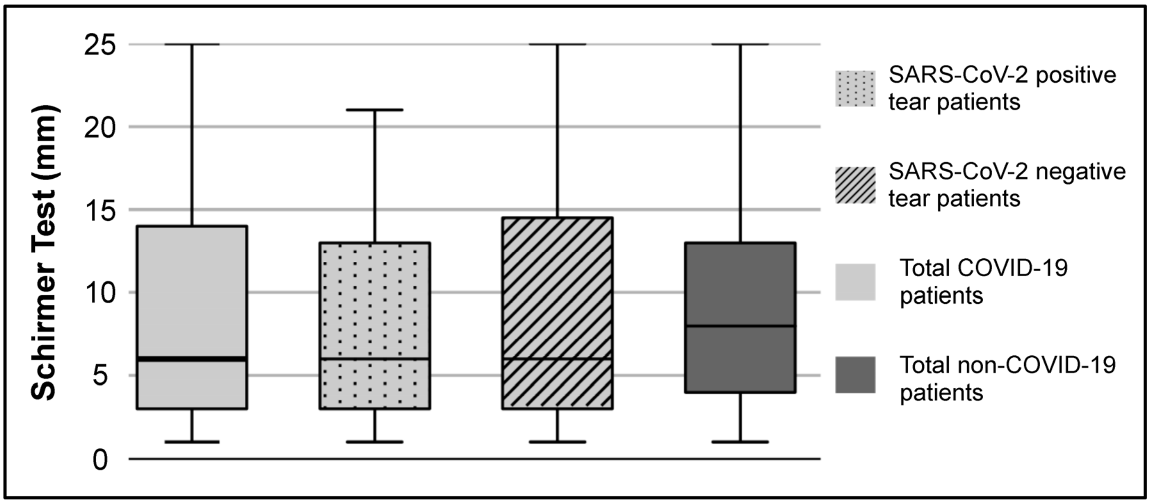

Figure S1. Schirmer test was not significantly different between COVID-19 and non-COVID-19 patients. Boxplot comparing Schirmer test results between patients with negative and positive SARS-CoV-2 in tear samples, total COVID-19 patients, and total non-COVID-19 patients.

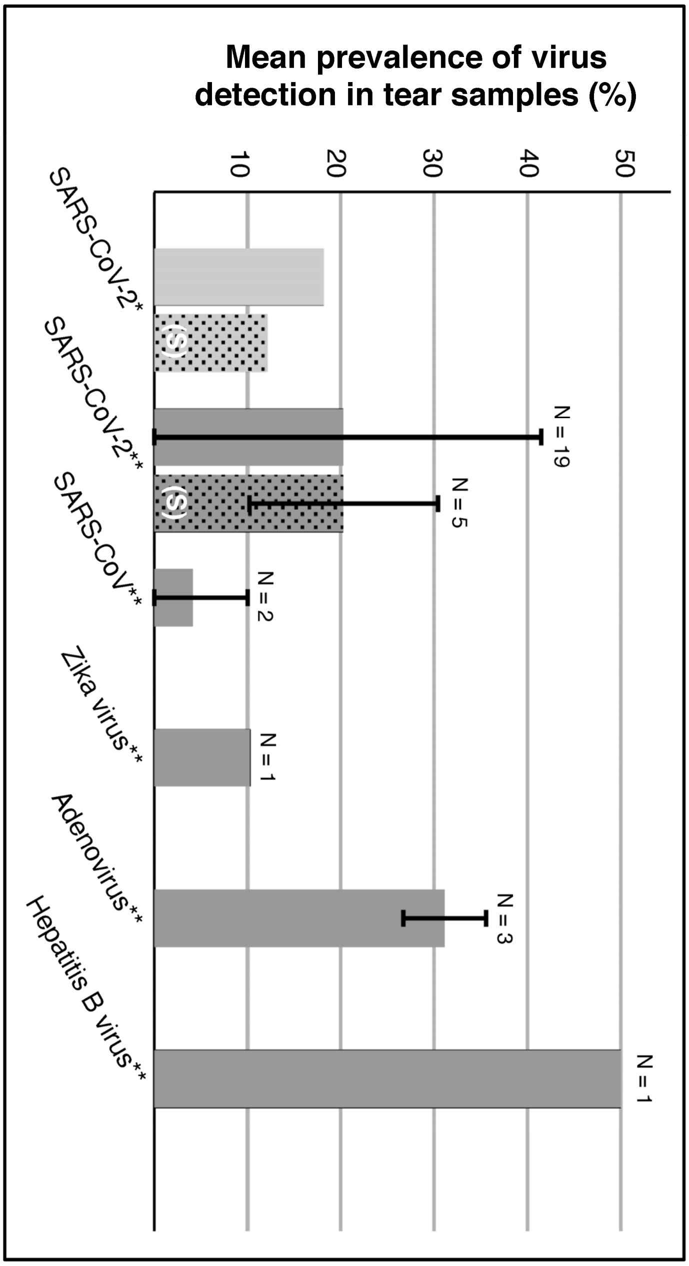

Figure S2. Systemic viruses show different detection prevalence in tear samples. The prevalence of SARS-CoV-2 detection in tear samples is compared to the detection of other systemic viruses in tear samples as reported in the literature. Only SARS-CoV-2 studies used Schirmer strips (S) for virus detection. N: number of studies included, *present study; **previous literature. Error bar: +/1 SD.

Table S1. Systemic symptoms in the confirmed COVID-19 group.

Symptoms	Possible Clinical Meaning	Prevalence in this study (n = 33)
Cough	May reflect upper and/or lower respiratory airway irritation.	66.7%
Dyspnea	Shortness of breath may show potential lower respiratory involvement.	60.6%
Fever	Measured body temperature >38°C. Potential generalized cytokine response.	36.4%
Myalgia	Muscle pain. May reflect a generalized inflammation and cytokine response.	15.2%
Asthenia	Fatigue, physical weakness and/or lack of energy. May reflect a generalized inflammation and cytokine response.	9.1%
Nausea	Stomach distress with an urge to vomit, may represent direct invasion of gastrointestinal epithelium and/or systemic inflammation.	6.1%
Diarrhea	Abnormal frequent evacuations with fluid stool. May represent direct invasion of gastrointestinal epithelium and/or systemic inflammation.	6.1%
Ageusia	Loss or impairment of taste sense may be related to damage and/or degradation of taste buds.	6.1%
Odynophagia	Painful swallowing may indicate direct inflammation of upper respiratory airway.	3.0%
Hyposmia	Partial loss of smell sense may show direct inflammation of upper respiratory airway with edema and/or tissue damage.	3.0%
Dysgeusia	Change or distortion in taste may be related to damage and/or degradation of taste buds and/or viral peripheral neurotropism.	3.0%
Anosmia	Complete loss of smell sense may show direct inflammation of upper respiratory airway with persistent olfactory epithelium inflammation.	3.0%

Table S2. Older patients also showed different comorbidities.

Age	Comorbidities
74	Cerebrovascular accident.
56	Systemic arterial hypertension, obesity, asthma, and chronic hepatopathy.
70	Systemic arterial hypertension, dyslipidemia, and arrhythmia.
60	Systemic arterial hypertension, obesity, dyslipidemia, and mitral insufficiency.
87	Systemic arterial hypertension and hypothyroidism.
78	Epilepsy.
69	Systemic arterial hypertension, obesity, and benign prostatic hyperplasia.
82	Systemic arterial hypertension, congestive heart failure, and dementia.
81	Diabetes Mellitus type 2, dementia, and prostate cancer.
76	Diabetes Mellitus type 2 and liver cancer.
74	Systemic arterial hypertension and chronic obstructive pulmonary disease.

Table S3. Patients who died were fully vaccinated, however they had three or more comorbidities. Table shows the factors that could be related to prognosis in all patients that died in the study. CCI: Charlson Comorbidity Index; CS: conjunctival swab; Ct: cycle threshold; RdRp: RNA-dependent RNA polymerase; SS: Schirmer strip.

Age	Sex	CCI	Comorbidities	SARS-CoV-2 Detection in tear samples (CS / SS)	Window between nasopharyngeal and tear tests (days)	Vaccine (doses)	CS Ct values: Nucleocapsid Envelope RdRp	SS Ct values: Nucleocapsid Envelope RdRp
56	M	21%	Systemic arterial hypertension, obesity, asthma, and chronic hepatopathy.	+ / −	4	CoronaVac (2)	38.23<br>39.36<br>40.48	>41<br>>41<br>>41
69	M	77%	Systemic arterial hypertension, obesity, and benign prostatic hyperplasia.	+ / +	1	CoronaVac (2)	25.69<br>24.72<br>28.18	25.69<br>24.72<br>30.33
82	F	0.0%	Systemic arterial hypertension, congestive heart failure, and dementia.	+ / +	1	CoronaVac (3)	34.83<br>34.39<br>>41	37.57<br>>41<br>>41
81	M	0.0%	Diabetes Mellitus type 2, dementia and prostate cancer.	− / −	2	CoronaVac (2)	>41<br>>41<br>>41	>41<br>>41<br>>41
74	M	77%	Systemic arterial hypertension and chronic obstructive pulmonary disease.	− / −	1	CoronaVac (2)	>41<br>>41<br>>41	>41<br>>41<br>>41
